# Comparing reference-based RNA-Seq mapping methods for non-human primate data

**DOI:** 10.1186/1471-2164-15-570

**Published:** 2014-07-07

**Authors:** Ashlee M Benjamin, Marshall Nichols, Thomas W Burke, Geoffrey S Ginsburg, Joseph E Lucas

**Affiliations:** Center for Applied Genomics, Department of Medicine, Duke University, Durham, North Carolina USA; Department of Electrical and Computer Engineering, Duke University, Durham, North Carolina USA

**Keywords:** RNA-Sequencing, Genomics, Mapping

## Abstract

**Background:**

The application of next-generation sequencing technology to gene expression quantification analysis, namely, RNA-Sequencing, has transformed the way in which gene expression studies are conducted and analyzed. These advances are of particular interest to researchers studying organisms with missing or incomplete genomes, as the need for knowledge of sequence information is overcome. *De novo* assembly methods have gained widespread acceptance in the RNA-Seq community for organisms with no true reference genome or transcriptome. While such methods have tremendous utility, computational cost is still a significant challenge for organisms with large and complex genomes.

**Results:**

In this manuscript, we present a comparison of four reference-based mapping methods for non-human primate data. We utilize TopHat2 and GSNAP for mapping to the human genome, and Bowtie2 and Stampy for mapping to the human genome and transcriptome for a total of six mapping approaches. For each of these methods, we explore mapping rates and locations, number of detected genes, correlations between computed expression values, and the utility of the resulting data for differential expression analysis.

**Conclusions:**

We show that reference-based mapping methods indeed have utility in RNA-Seq analysis of mammalian data with no true reference, and the details of mapping methods should be carefully considered when doing so. Critical algorithm features include short seed sequences, the allowance of mismatches, and the allowance of gapped alignments in addition to splice junction gaps. Such features facilitate sensitive alignment of non-human primate RNA-Seq data to a human reference.

**Electronic supplementary material:**

The online version of this article (doi:10.1186/1471-2164-15-570) contains supplementary material, which is available to authorized users.

## Background

For the past decade, microarray gene expression data has revolutionized all areas of life science - allowing quantification of thousands of genes in several samples simultaneously, and paving the way for countless research studies. With the advent of RNA-Seq data, researchers now have the ability to perform untargeted gene expression analysis via next generation sequencing (NGS) technology, obtaining qualitative sequence information as well as quantitative gene expression data. RNA-Seq provides a comprehensive gene expression profile of each sample – with the potential to quantify and annotate all genes and isoforms. This untargeted approach proves particularly useful when quantifying gene expression in polymorphic cell lines and in organisms with a nonexistent or provisional reference genome where the sequence of features to be quantified is unknown [1, 2]. Due limited availability of genomic resources for under-characterized species, RNA-Seq is a popular method of choice for transcriptome analyses. We present a comparison of reference-based mapping methods for RNA-Seq data originating from Non-Human Primates (NHPs), and their implications in downstream differential expression analysis.

### Mapping and assembly

RNA-Seq obtains gene expression estimates by assigning next-generation sequencing (NGS) reads to transcripts, either by mapping to a reference sequence, or assembling into contiguous stretches of sequence data (contigs) utilizing overlapping sequence amongst the reads themselves. These methods are termed reference-based alignment approaches, and *de novo* assembly approaches, respectively. Reference-based alignment and *de novo* assembly each have advantages and disadvantages. The optimal strategy likely depends on the experimental design and available genomic and computational resources. Reference-based approaches are far less computationally intensive than *de novo* approaches, and are well-suited for detection of low abundance transcripts [3]. However, reference-based mapping relies on the availability and accuracy of a reference sequence. Mapping approaches must also handle reads with more than one potential mapping location. Such uncertainty can arise from paralogous gene families, repetitive sequences, and shared exons of alternatively spliced transcripts [4]. Conversely, *De novo* assemblers have the advantage of not requiring a reference sequence. This allows discovery of transcripts not present in a reference genome or transcriptome. *De novo* assemblers overcome challenges caused by mapping uncertainty, as well as long introns that may be missed by reference-based mapping methods. While the freedom from a reference sequence is appealing, *de novo* assemblers require large amounts of computational resources and assembly time. In addition, deeper sequencing is needed for adequate *de novo* transcript assembly than is required by reference-based approaches [3]. *De novo* assembly can be very useful for organisms with no reference genome, but due to its computational demands is most commonly used in cases of small genomes such as bacteria, archaea, and lower eukaryotes [3]. In the case of large and complex transcriptomes, such as plants and mammals, reference-based mapping methods can overcome the computational and isoform-resolving challenges faced by *de novo* assemblers [3]. In general, reference-based mapping approaches are the appropriate choice when a reliable reference genome exists. The choice becomes less clear when working with complex mammalian species with little to no genomic resources, such as under-characterized NHPs.

We propose the use of a human reference genome (or transcriptome) for reference-based mapping and expression quantification of NHP RNA-Seq data. An elegant study by Hornett et al. [2] evaluated the utility of divergent species gene sets for annotation of *de novo* assembly. When utilizing reference gene sets from divergent species, the authors found little bias in expression levels and strong correlation in gene expression up to approximately a 100 million year window. More divergent species (greater than 100 million years apart) suffered from incorrect assignment of assembled contigs to genes. The authors found little difference in the number of genes having contigs assigned, when using chimpanzee, orangutan, macaque, or marmoset vs. human. In addition, the authors compared the use of *de novo* assembled transcriptomes to mapping directly to the reference predicted gene set for quantifying gene expression. When comparing the mapping of the reads directly to the predicted gene set vs. the *de novo* assemblies, the authors found that mapping to the gene set recovered expression data for more genes, and that expression levels were strongly correlated within gene sets detected by both methods [2].

In this manuscript, we examine the mapping efficacy and utility for differential expression analyses of various reference-based approaches when the reference sequence originates from a closely related species. Specifically, we utilize the human genome and transcriptome as a reference for non-human primate RNA-Seq data from yellow baboons, *Papio cynocephalus*. We compare different reference-based mapping approaches – one representative method from four different mapping method categories [5] to map to the reference genome, and two of these methods to map to the reference transcriptome as well.

### Reference-based mapping methods

Reference-based alignment methods utilize the sequence for each read (and it’s mate in paired-end data) to find potential mapping locations by exact match or scoring sequence similarity. Mapping locations indicate transcripts of origin, and the number of reads originating from a given transcript inform on how much of the transcript was present in the sample. We briefly describe four categories of reference-based mapping methods, and subsequently show the results of one representative method from each category when mapping RNA-Seq reads from yellow baboon to a human reference. A summary of the four categories and the representative methods tested in this study is shown in Table 1.

**Table 1:** **Reference-based mapping methods overview - summary of the four reference-based mapping method categories compared in this study**

Method	Reference	Method subcategory	Representative	Notes
category			method	
Unspliced	Genome or	Burrows-Wheeler	Bowtie2*	Index reference sequence, Rapidly look up candidate mapping loci.
Aligners	Transcriptome	Transform Method		Typically faster and less sensitive than Seed Methods.
		Seed Method	Stampy	Align short subsequences of reads to find candidate mapping loci,
				Narrow candidates by extending alignments. Typically slower and
				more sensitive than Burrows-Wheeler Transform Methods.
Spliced	Genome	Exon First Method	TopHat2	Align whole reads with Unspliced Aligners, Search for spliced
Aligners				alignments in remaining reads. Typically faster and less sensitive
				than Seed and Extend Methods.
		Seed and Extend Method	GSNAP	Align short subsequences of reads to find candidate mapping loci,
				Narrow candidates by extending alignments. Typically slower and
				more sensitive than Exon First Methods.

^*^Bowtie2 may be considered a hybrid BWT-Seed Method, as multiple substrings are taken from each read for the BWT lookup of candidate mapping loci, and the alignment at each candidate loci is extended.

The first major category has been referred to as “unspliced read aligners”, which align reads to a reference without allowing large gaps such as those arising from reads spanning exon boundaries, or splice junctions [6–12]. Unspliced aligners may be used to align reads to a reference transcriptome or a reference genome. Aligning to a reference transcriptome alleviates the need to handle splice junctions, but is limited to the analysis of known transcripts. When utilizing unspliced read aligners to map to a reference genome, reads may be mapped to potentially novel exons, however reads spanning splice junctions are likely to remain unmapped. Unspliced read aligners are generally divided into two subcategories based on their methodology -“seed methods” and “Burrows-Wheeler transform (BWT) methods” [5]. Seed methods align short subsequences, or seeds, from each read to a reference, requiring a perfect match in the seed subsequence. More sensitive alignment methods are then used to eliminate candidate regions where seeds cannot be extended to full read alignments. The unspliced seed method we chose to test is Stampy [11]. BWT methods create a Burrows-Wheeler index of the reference sequence and efficiently search for perfect matches. Mismatches may be allowed with an exponential increase in computational complexity. In general, Burrows-Wheeler transform methods are faster than seed methods, but seed methods provide increased sensitivity [3]. The unspliced BWT method we chose to test is Bowtie2 [12]. A similar analysis reported that when the true reference transcriptome is available BWT methods are faster with minimal differences in alignment specificity. When the reference transcriptome of a distant species is available, seed methods result in large increases in sensitivity [5]. These increases in sensitivity have also been observed when aligning reads to polymorphic regions [13].

Methods in the unspliced read aligner category are either limited by their inability to handle reads spanning splice junctions when mapping to a reference genome, or limited to known exons and splice sites when mapping to a reference transcriptome. The second major category of mapping methods, “spliced aligners”, align reads to the whole genome, with intron-spanning reads requiring large gaps. Spliced aligners accommodate junction-spanning reads by splitting them up into smaller segments and determining the best match based on alignment scores and known di-nucleotide splice signals [14–18]. The spliced aligners also fall into two major categories based on their methodology - “exon-first” methods and “seed-and-extend” methods. Exon-first methods begin by mapping whole reads to the genome using unspliced read aligners, and then search for spliced alignments with the remaining reads. Exon-first approaches are efficient, but may miss true spliced alignments when an unspliced alignment is available in a pseudogene [5]. Seed-and-extend methods break reads into seeds which are mapped to the genome, and much like seed-based unspliced aligners, candidate mapping locations are examined with more sensitive alignment methods. Iterative extension and merging of initial seeds is performed to determine the spliced alignment. As with unspliced aligners, seed-and-extend methods are slower, but more sensitive, and show improved performance when mapping reads from polymorphic samples [5]. The exon-first and seed-and-extend methods we chose to test are TopHat2 [19], and GSNAP [18], respectively.

After mapping to a reference genome, a transcriptome reconstruction step is required to appropriately assign reads to transcipts. Aligned reads spanning splice junctions are “connected”, and read counts to various isoforms of each gene are reported. This is accomplished by building a graph to represent all possible isoforms of an expressed feature. Different paths through the graph represent individial isoforms [20, 21].

We present a comparison of reference-based mapping methods for RNA-Seq data having no true reference, but that of a closely related model organism. We assess the various methods using mapping rates, mapping locations, correlation of gene expression, as well as the utility of the data for differential expression analyses. To better understand the differences between the methods, we examine more closely the default behaviors of the four mapping methods used.

Bowtie2 is a BWT-based unspliced aligner, with the recent addition of supported gapped alignments. This method may actually be considered a combination BWT and seed unspliced mapper. Bowtie2 extracts multiple substrings or seeds from each read and aligns them using a BWT approach with no gaps, then extends alignments using a Smith-Waterman-like scoring scheme. By default, seeds are 22 bp and no mismatches are allowed within the seed. Base call quality scores are incorporated by assigning more severe mismatch penalties at high-quality read positions. Gap initiation and extension penalties are also utilized, while the number and lengths of gaps within extended alignments are not restricted. Bowtie2 does not guarantee that the alignment reported is the best in terms of alignment score, and when there is more than one potential mapping location of equal score, one reported location is selected at random.

Stampy is a seed-based unspliced aligner that uses a hash table to store the locations of 15-mers in the reference sequence. For each read, candidate alignment locations are identified with a hash lookup of the 15-mers in the read, allowing for one mismatch. Candidate mapping locations are screened for sequence similarity with the read, and then full alignments are attempted at each remaining candidate location. As with Bowtie2, Stampy also considers base quality calls, and allows gaps in this alignment step. Stampy also allows the use of BWT as a “pre-mapping” step to increase speed, however the manual does not recommend this for paired-end data. For this reason, and for method-comparison purposes, we did not use the BWT option. Stampy uses a Bayesian probabilistic model to represent mapping quality, and reports the single best alignment location.

TopHat2 is an exon-first spliced read aligner that uses Bowtie2 as a base algorithm. Like the original TopHat, potential splice sites are detected within candidate alignment locations, and used in a subsequent step to align reads spanning exon-junctions. TopHat2 first maps to the transcriptome with Bowtie2. Remaining whole reads are then mapped to the reference genome, and then spliced alignments are attempted. Most of the default Bowtie2 parameters when run within Tophat2 are the same as the default standalone Bowtie2 parameters, with the exception of seed length and intervals between seeds. TopHat2 seeds within Bowtie2 are 20 bp, and the interval between seeds is longer. TopHat2 reduces alignment to pseudogenes by aligning reads preferentially to genes within provided annotation. This use of annotation by TopHat2 has been shown to increase sensitivity and accuracy of mapping. We provided TopHat2 with annotation information for this purpose. In addition to gapped alignment in the Bowtie2 step, TopHat2 also allows indels in the spliced alignment detection step.

GSNAP is a seed-extend spliced aligner that allows for long and even chromosome spanning gaps, likely resulting from gene-fusion events. GSNAP uses all 12-mers in the read to identify candidate mapping locations, not favoring positions within short reads. Alignments are extended at candidate loci, requiring that the read has a consecutive stretch of 14 nucleotides exactly matching the reference sequence. GSNAP allows multiple mismatches and long indels, but only allows one splice or indel per read. Splicing is identified in two different ways - searching surrounding sequence for splice signals, or a user-provided set of known exon-intron boundaries.

Also worth noting is each methods’ default handling of reads mapping to multiple locations. With the default parameters, TopHat2 reports the single alignment having the best alignment score. If there are multiple locations with the same optimal alignment score, TopHat2 will report up to 20 of these results. Bowtie2 by default performs a non-exhaustive search for distinct valid alignments, and then reports the single best result of the identified alignments. If there are multiple alignments with the same alignment score, a single result is selected at random. Because the search is not exhaustive, Bowtie2 does not guarantee that the alignment reported is the absolute best in terms of alignment score. Similar to Bowtie2, Stampy reports the single best alignment, selecting one result at random when more than one alignment shares the best mapping quality. GSNAP reports up to 100 valid mapping loci based on match, mismatch, and gap thresholds. As with the matching parameters, all of the reporting parameters of these mapping tools may be adjusted.

## Results and discussion

We mapped RNA-Seq reads from 57 yellow baboon (*Papio cynocephalus*) peripheral blood samples to a human reference using four different mapping methods, two of which were used to map to the reference genome and the reference transcriptome. Specifically, the six different techniques included Bowtie2 [12], Stampy [11], TopHat2 [19], and GSNAP [18] for mapping to the reference genome, and Bowtie2 and Stampy for mapping to the reference transcriptome. We then assessed the utility of each mapping method with mapping rates, base mismatch rates, mapping locations, detected transcripts, correlation of gene expression estimates, and differential expression analysis results. The 57 baboon samples were taken from 12 different animals at 5 time points, where the phenotype of interest is clinical bacterial pneumonia infection (see Methods and Table 2 for more details). When mapping RNA-Seq reads from a distant NHP species to human reference sequence, we see differences in the utility of various mapping methods with respect to mapping rates, detected genes, correlation of expression values, and differentially expressed genes.

**Table 2:** **Study participants and RNA sequencing read pairs in millions before, and after pre-mapping quality control**

Participant number	Bacterial dose (CFU)	0 Hours	6 Hours	24 Hours	48 Hours	168 Hours
1	0	37.0, 36.2	44.3, 42.9	33.8, 32.9	35.5, 34.8	32.6, 32.0
2	0	34.0, 33.4	31.8, 31.1	34.6, 34.0	34.0, 32.9	35.8,35.2
3	0	31.3, 30.6	37.2, 36.5	31.1, 30.5	◯	36.8, 36.1
4	10^6^	37.8, 37.0	31.5, 31.0	36.5, 35.8	31.1, 30.6	35.8, 34.9
5*	10^7^	31.2, 30.5	35.4, 34.6	37.8, 36.9	34.0, 33.4	35.8, 35.1
6*	10^8^	36.3, 35.5	32.6, 31.7	36.1, 35.3	34.5, 33.8	34.7, 34.0
7*	10^8^	38.0, 37.2	32.4, 31.5	◯	31.0, 30.4	37.6, 36.9
8	10^8^	35.9, 35.2	35.3, 34.5	◯	31.9, 31.3	32.7, 32.0
9*	10^9^	36.2, 35.5	32.6, 32.0	36.6, 35.8	33.6, 32.9	34.8, 34.1
10*	10^9^	34.5, 33.8	33.3, 32.8	37.6, 36.6	36.2, 35.5	34.3, 33.7
11*	10^9^	35.4, 34.7	35.5, 34.7	31.9, 31.3	35.6, 34.5	30.1, 29.5
12*	10^9^	36.2, 35.4	35.4, 34.8	34.8, 34.1	30.9, 30.3	35.2, 34.6

^*^Participant met clinical criteria for pneumonia at 48 hours. **◯** - Failed RNA quality test (was not sequenced).

### Mapping statistics

**Figure 1:**
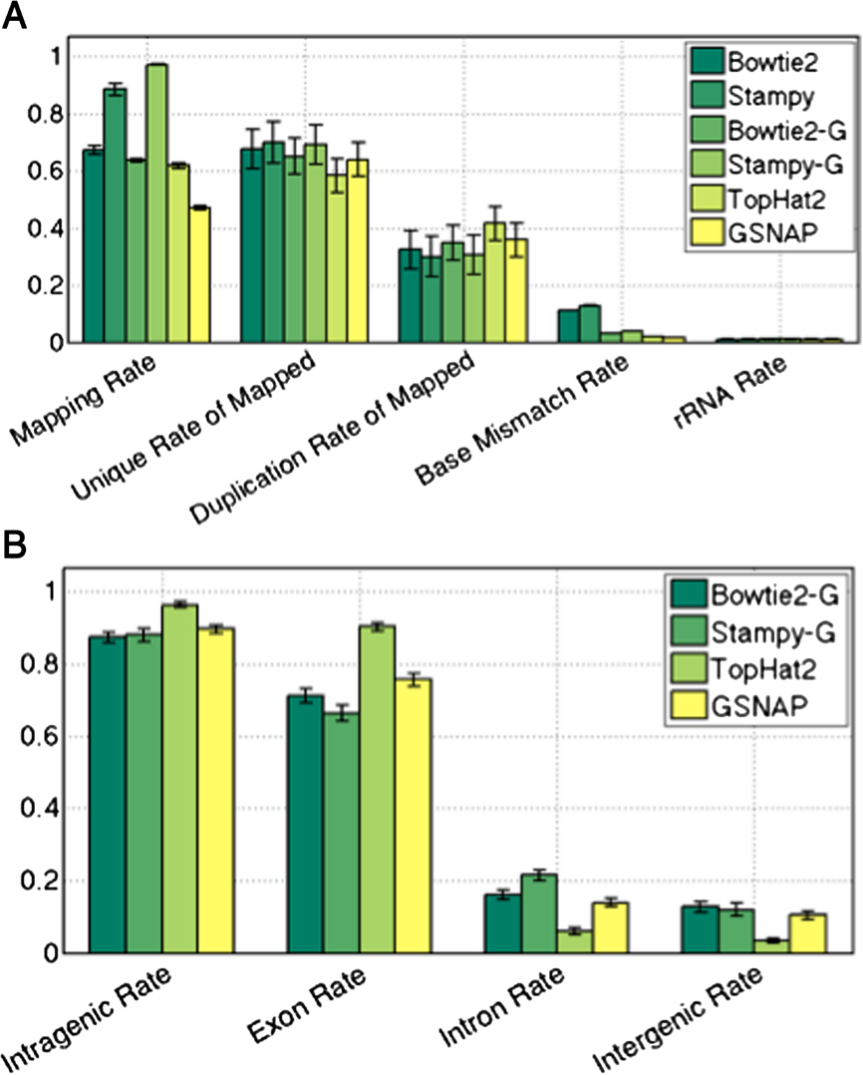
**Mapping Statistics for Reference Transcriptome and Reference Genome Methods.** Panel **A** shows the mapping, unique, duplication, base mismatch, and rRNA rate for each of the mapping methods. Error bars show plus/minus one standard deviation. Mapping rate is computed as mapped reads divided by total reads, unique rate is computed as unique mapped reads divided by mapped reads, duplication rate is computed as duplicate mapped reads divided by mapped reads, base mismatch rate is computed as the number of bases not matching the reference divided by the number of aligned bases, and rRNA rate is computed as the number of reads mapping to ribsomal RNA divided by the total reads. Panel **B** shows the mapping locations for the two reference genome mapping methods. Each value is computed as the number of reads mapping to a type of region divided by the total reads mapped.

For each of the mapping methods, we computed the average and standard deviation of several mapping statistics across the 57 samples. Table 3 shows a summary of these mapping statistics. We first examine overall mapping rates and locations of the four methods, illustrated in Figure 1. Examining the overall mapping rates, we see that GSNAP obtains the lowest mapping rates, ranging from 0.4497 to 0.4857. This is likely due to the 14 nucleotide exact match requirement, and the single gap or splice restriction. Any reads spanning exon junctions that would align well with an additional small gap on either side of the intron will not be considered. Other reads mapping to divergent regions may not have a continuous stretch of 14 conserved nucleotides. TopHat2 was reported to be more sensitive and accurate than GSNAP [19], which was of similar sensitivity as the original TopHat [18]. GSNAP was found to perform poorly on short-anchored reads (small “anchor” on either end of a splice junction) [19]. TopHat2 and GSNAP were found to perform similarly on single-end reads with small indels (1-3 bp), but GSNAP performed better on indel alignments with paired-end reads [19]. In this study TopHat2 obtains significantly higher mapping rates ranging from 0.5940 to 0.6365. Bowtie2 mapping to the reference genome (Bowtie2-G) obtains higher still mapping rates of 0.6227 to 0.6530. When mapping to the reference transcriptome, Bowtie2 obtains slightly higher mapping rates of 0.6269 to 0.7052. We see slightly lower mapping rates with TopHat2 than Bowtie2, regardless of the fact that Bowtie2 is the underlying algorithm of TopHat2. As previously mentioned, the only difference in default parameters is the seed length and interval between seeds. TopHat2 runs Bowtie2 with a shorter seed, which would suggest increased sensitivity, but a longer interval between seeds, which would lead to decreased sensitivity. Because the interval between seeds is longer, less seeds are used to identify candidate mapping locations, and with the default of no mismatches allowed within a seed some correct alignment loci would not be considered. Stampy achieves the highest mapping rates ranging from 0.8199 to 0.9358 when mapping to the reference transcriptome, and 0.9640 to 0.9772 when mapping to the reference genome (Stampy-G). This is likely due to the shorter seed length, and the allowance for a single mismatch in seeds, allowing more candidate loci to be considered. All increases in mapping rates were significant.

**Table 3:** **Summary of mapping metrics results for the compared reference-based methods**

Reference		Transcriptome		Genome			
Comparison Metric	Value	Bowtie2	Stampy	Bowtie2-G	Stampy-G	TopHat2	GSNAP
Mapping Rate	Mean	0.673	0.885	0.638	0.972	0.618	0.472
	SD	0.016	0.021	0.006	0.003	0.008	0.007
Base Mismatch Rate	Mean	0.113	0.128	0.033	0.039	0.020	0.017
	SD	0.001	0.002	0.001	0.001	0.000	0.000
Intragenic Mapping Rate	Mean	1	1	0.873	0.880	0.964	0.896
	SD	N/A	N/A	0.014	0.018	0.007	0.012
Intergenic Mapping Rate	Mean	0	0	0.127	0.112	0.034	0.104
	SD	N/A	N/A	0.014	0.018	0.007	0.012
rRNA Mapping Rate	Mean	0.011	0.011	0.112	0.122	0.011	0.011
	SD	0.003	0.003	0.003	0.003	0.003	0.003
Detected Transcripts	Mean	31615	37585	28128	29175	31338	27264
	SD	803.209	815.801	926.053	887.524	807.139	945.805
Correlation Between Baseline Samples	Mean	0.977	0.983	0.981	0.978	0.981	0.981
	SD	0.007	0.005	0.004	0.005	0.005	0.005
Differentially Expressed Genes	Number	2043	2122	1797	2017	1953	1545

SD - Standard Deviation.

We also observe higher base mismatch rates when mapping to the reference transcriptome. This may be partly due to the fact that the default settings of Bowtie2 and Stampy are more sensitive, and so reads may be successfully mapped to more divergent regions. Another possible contributing factor is the presence of splice variants in baboon not present in human. The human reference transcripts may contain additional or missing exons with respect to baboon, causing mapped reads that span the true exon junction to have a high mismatch rate for a short stretch of sequence. Unique, duplication, and rRNA rates are all similar across the mapping methods.

We also examined the mapping locations - intergenic and intragenic (exonic and intronic). Mapping to the reference transcriptome obtains an intragenic mapping rate of 1, simply due to the nature of the mapping procedure. When mapping to the reference genome, we see that Bowtie2, Stampy, and GSNAP all obtain a significantly higher intergenic and intronic mapping rates than TopHat2, ranging from 0.1015 to 0.186 (Bowtie2-G), 0.0917 to 0.2011 (Stampy-G), 0.0856 to 0.1537 (GSNAP), and 0.0248 to 0.0654 (TopHat2). Conversely, TopHat2 obtains a significantly higher exonic mapping rates ranging from 0.8705 to 0.9258 compared to 0.6519 to 0.7722 for Bowtie2-G, 0.5927 to 0.7285 for Stampy, and 0.7023 to 0.7938 for GSNAP. This is likely due to TopHat2’s preferential mapping to the reference transcriptome prior to exploration of genomic locations.

### Detected transcripts

**Figure 2:**
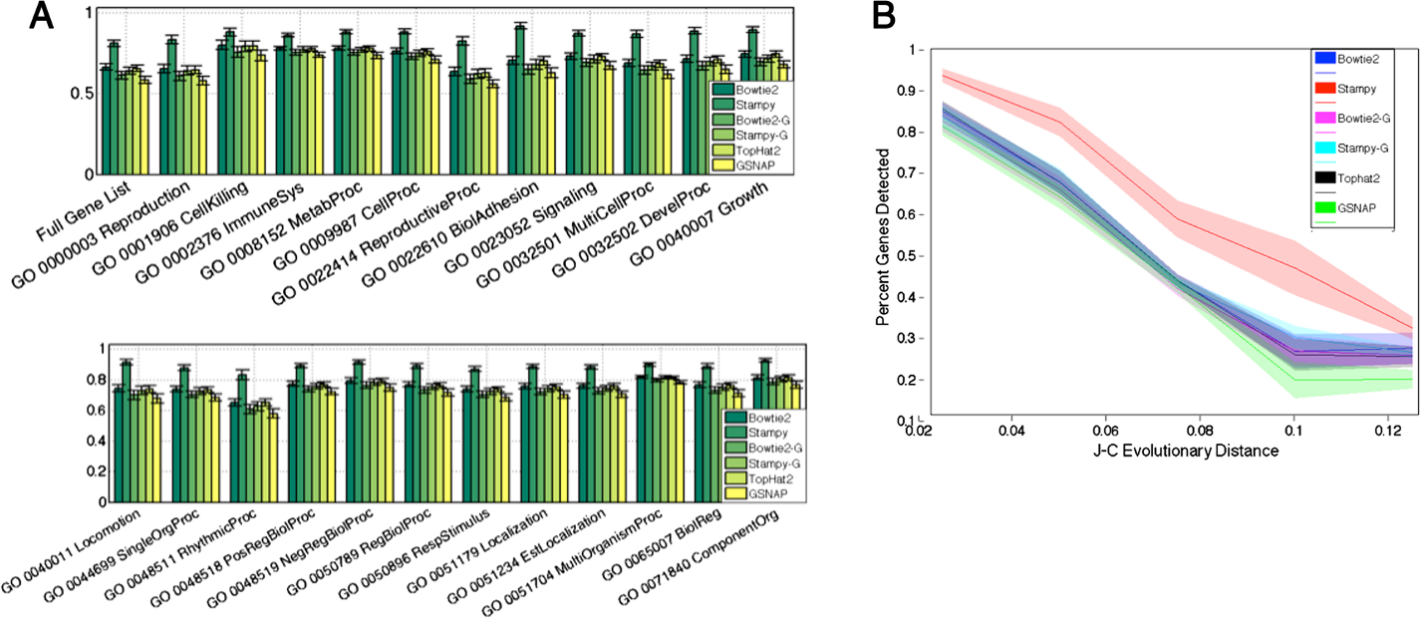
**Detected Genes by Function and Evolutionary Distance.** Panel **A** shows the mean and standard deviation of percent detected genes (computed as detected genes within a list divided by the number of genes within the list) for the full gene set, and 23 different Gene Ontology Biological Process groupings. Panel **B** shows the mean and standard deviation of percent detected genes at increasing evolutionary distance strata.

Using RNA-SeQC version 1.1.7 [22], we computed the number of known human transcripts detected by each method. RNA-SeQC defines a transcript as detected if at least 5 reads map to an exon region. We found that all methods detect significantly more known transcripts than GSNAP, which ranges from 25,069-28,585. Bowtie2-G detected significantly more transcripts than GSNAP, ranging from 25,921-29,435. Stampy-G detected significantly more transcripts than GSNAP and Bowtie2-G, ranging from 27,044-30,459. Stampy-G was followed by Bowtie2 and TopHat2, ranging from 29,631-32,817, and 29,427-32,556, respectively. The difference in detected transcripts between Bowtie2 and TopHat2 was not significant. Finally, Stampy detected significantly more transcripts than all other methods, ranging from 35,308-39,234. We do not see 100% of transcripts represented, but that is to be expected. The transcripts present in a single tissue type will not contain an organisms full repertoire of transcripts [2, 23]. There were a total of 44,312 human transcripts in our annotation set (iGenomes UCSC hg19, Ensemble annotation). We also examined the detected genes, collapsing all splice variants, in functional groupings determined by Gene Ontology [24] annotations. Panel A of Figure 2 shows the percent of genes detected within each gene list. We observe little differences in the ability of each mapping method to detect genes in different functional groups compared to the full gene set. It is worth noting that for some functional groups, the differences seem less or more pronounced. For example, Stampy’s increased sensitivity seems less pronounced within immune system genes. This may be due to the nature of our samples - we might expect immune system genes to be highly expressed. Similarly, we examined the ability of each mapping method to detect genes at varying evolutionary distances. Using *Papio anubis* RefSeq genes as a surrogate for *Papio cynocephalus*, we identified human orthologs, computed Jukes-Cantor evolutionary distance, and examine the percent of genes detected within evolutionary distance strata (see Methods). Panel B of Figure 2 shows the mean and standard deviation for percent of genes detected at increasing evolutionary distances. While this is a small set of genes, we see an approximately linear decrease in percent genes detected as evolutionary distance increases, up until the highest evolutionary distances, where the difference in sensitivity is minimized as all methods lose the ability to detect genes. We still see that Stampy detects the most genes, followed by Bowtie2, TopHat2, Stampy-G, Bowtie2-G, and GSNAP.

### Correlation of gene expression

**Figure 3:**
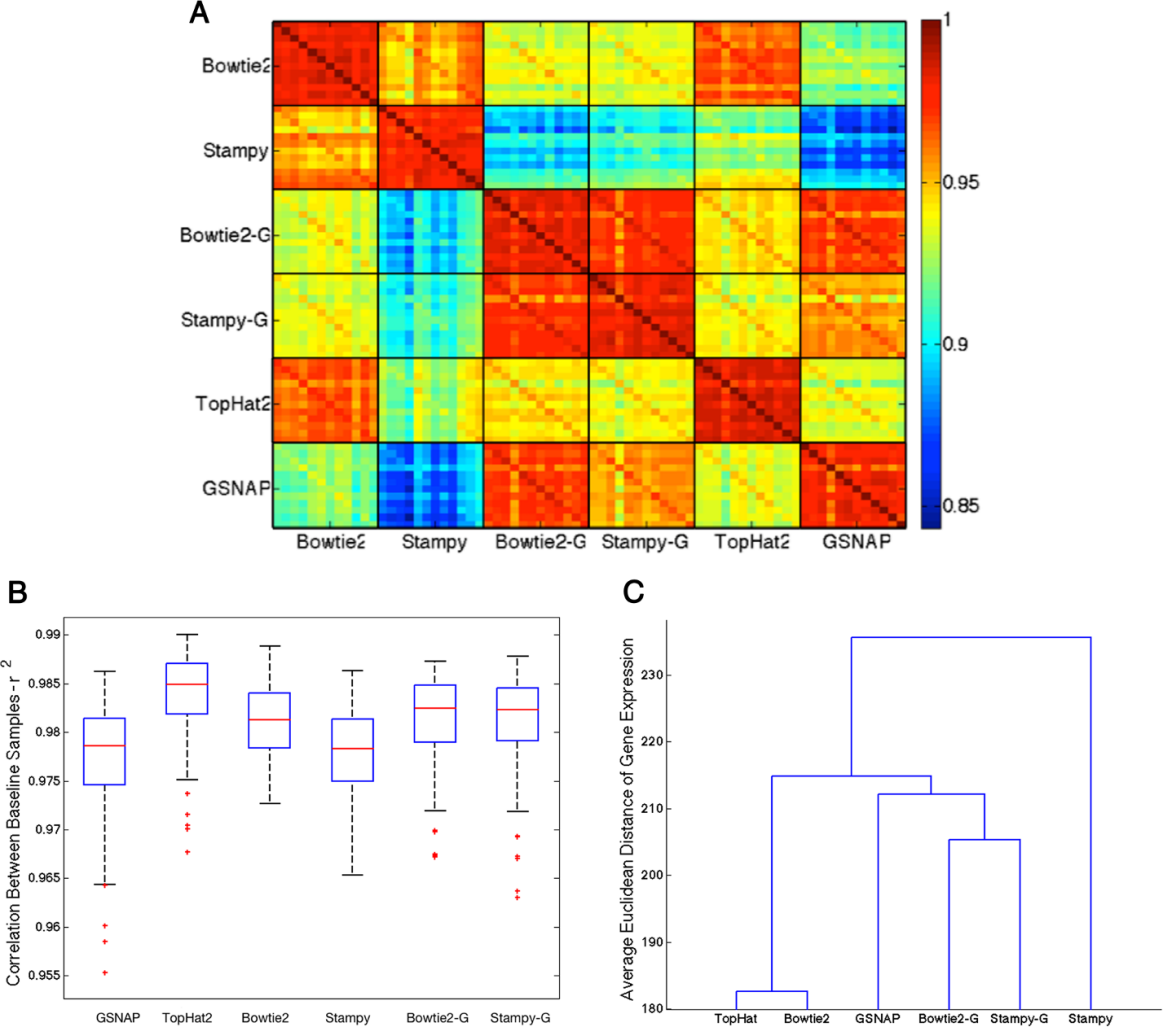
**Correlation of Gene Expression.** Panel **A** shows a heat map of all pairwise Pearson correlations between gene expression of each baselines (0 hours) sample computed with the results of each of the mapping methods. Panel **B** shows boxplots of the correlations between gene expression of baseline samples (0 hours), within each method. Panel **C** shows the dendrogram of gene expression using the average Euclidean distance between gene expression estimates for each sample.

We examined correlations of normalized and log-transformed gene expression between baseline samples within the same mapping method, and correlations between normalized and log-transformed gene expression levels computed with results from the different mapping methods. Panel A of Figure 3 shows a heat map of the Pearson correlations between all biological replicates of baseline samples, for all methods. We see strong correlation between samples for all methods (the large blocks along the diagonal). Similarly, Panel B shows boxplots of these correlations. These correlation coefficients are very strong, with none falling below 0.9314, and the mean of each method above 0.97. The Spearman correlation coefficients were also computed, yielding similar results with slightly lower correlation coefficients overall. These results are shown in Additional file 1: Figure S1 and S2.

We also computed the Pearson correlation between the different methods for identical samples. This is illustrated by the blocks off the diagonal in Panel A of Figure 3. Bowtie2-G and Stampy-G had the highest correlations, ranging from 0.9805 to 0.9877. GSNAP and Stampy had the lowest correlations, ranging from 0.8690 to 0.9047. As performed in the above analysis,the Spearman correlation coefficients were also computed. When examining correlations between different methods for identical samples we also observed similar results with slightly lower correlation coefficients overall. These results are shown in Additional file 1: Figure S1.To further illustrate the concordance in gene expression estimates obtained with each mapping method, we constructed a dendrogram, computing the Euclidean distance in gene expression between methods for each of the baseline (0 hours) samples, and then averaging the distances. Examining the dendrogram in Panel C of Figure 3, we observe TopHat2 and Bowtie2 have the shortest distance between gene expression estimates, followed by GSNAP, Bowtie2-G, and Stampy-G, and finally Stampy. These results are in accordance with the correlations shown in Panel A of Figure 3. The weakest correlations are seen between the most and least sensitive methods (with respect to detecting human genes), Stampy and GSNAP, respectively. This difference is less pronounced for methods of comparable sensitivity such as Bowtie2 and TopHat2. For the less sensitive methods, it is possible that reads from divergent regions of transcripts are not successfully mapped, affecting expression estimates.

### Differential expression analysis

**Figure 4:**
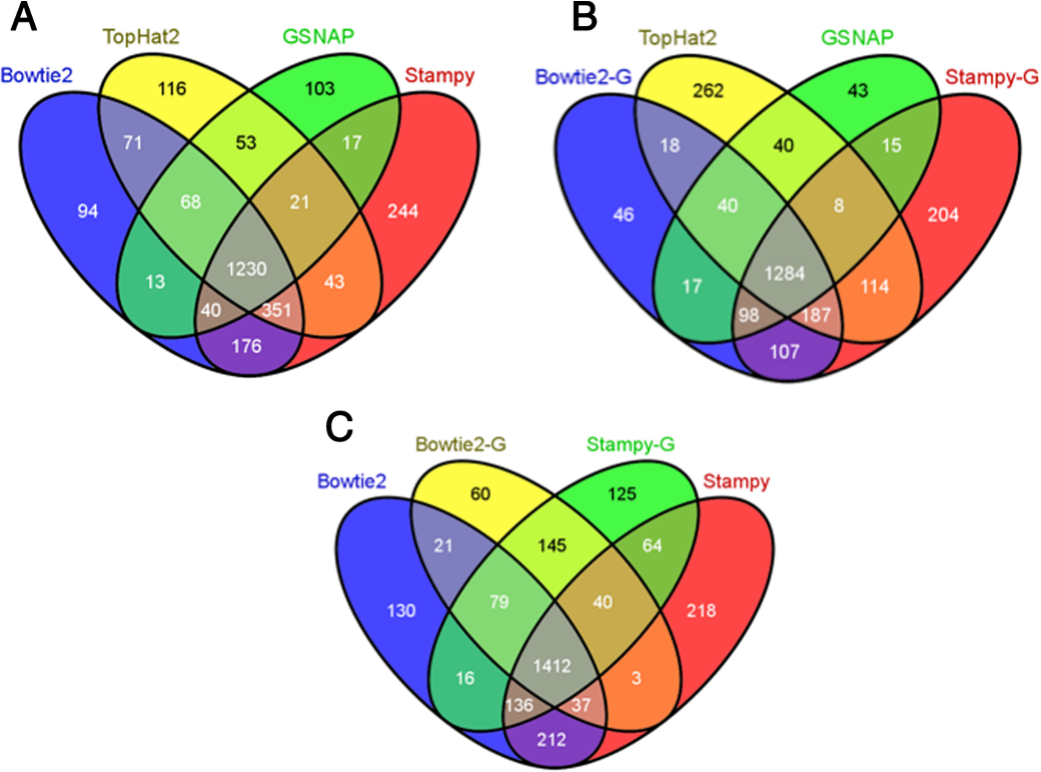
**Differential Expression Overlap.** Panel **A** shows a venn diagram of the differentially expressed genes found using the transcriptome mappings of Bowtie2 and Stampy, and the genome mappings of TopHat2 and GSNAP. Panel **B** shows a venn diagram of the differentially expressed genes found using the genome mappings of Bowtie2, Stampy, TopHat2, and GSNAP. Panel **C** shows a venn diagram of the differentially expressed genes found using the transcriptome mappings of Bowtie2 and Stampy, and the genome mappings of Bowtie2 and Stampy.

We used edgeR to identify differentially expressed genes between healthy and sick samples from the expression estimates computed with each mapping method. Sick participants were considered to be animals meeting clinical criteria for bacterial pneumonia infection, at the time of maximal symptoms (48 hours). We define healthy samples as all baseline samples, as well as the control doses (0 CFU) at the maximal symptom timepoint of 48 hours. Table 2 illustrates the samples collected from each study participant, and their clinical status. In the context of this table, our differential expression analysis compared the 0 hour timepoint of all study participants and the 48 hour timepoint of participants 1 and 2, with the 48 hour timepoint of participants 5, 6, 7, 9, 10, 11, and 12. Figure 4 shows three 4-way venn diagrams of the overlap in significant differentially expressed genes after Bonferonni correction, resulting from the different mapping methods. There are a large number of differentially expressed (DE) genes shared by the methods. Many of the other DE genes are shared between 3 methods. Overall, GSNAP results in the fewest DE genes, followed by Bowtie2-G, and TopHat. Stampy-G, Bowtie2, and Stampy yield the most differentially expressed genes, which is not surprising given their higher number of overall detected transcripts. The ability to detect differential gene expression will likely be less dependent on mapping method, as the ability to map reads with a single method will be constant across samples. More sensitive methods, however, may identify more differentially expressed genes simply because these genes are detected by these methods, and not others.

### Read counts by evolutionary distance

**Figure 5:**
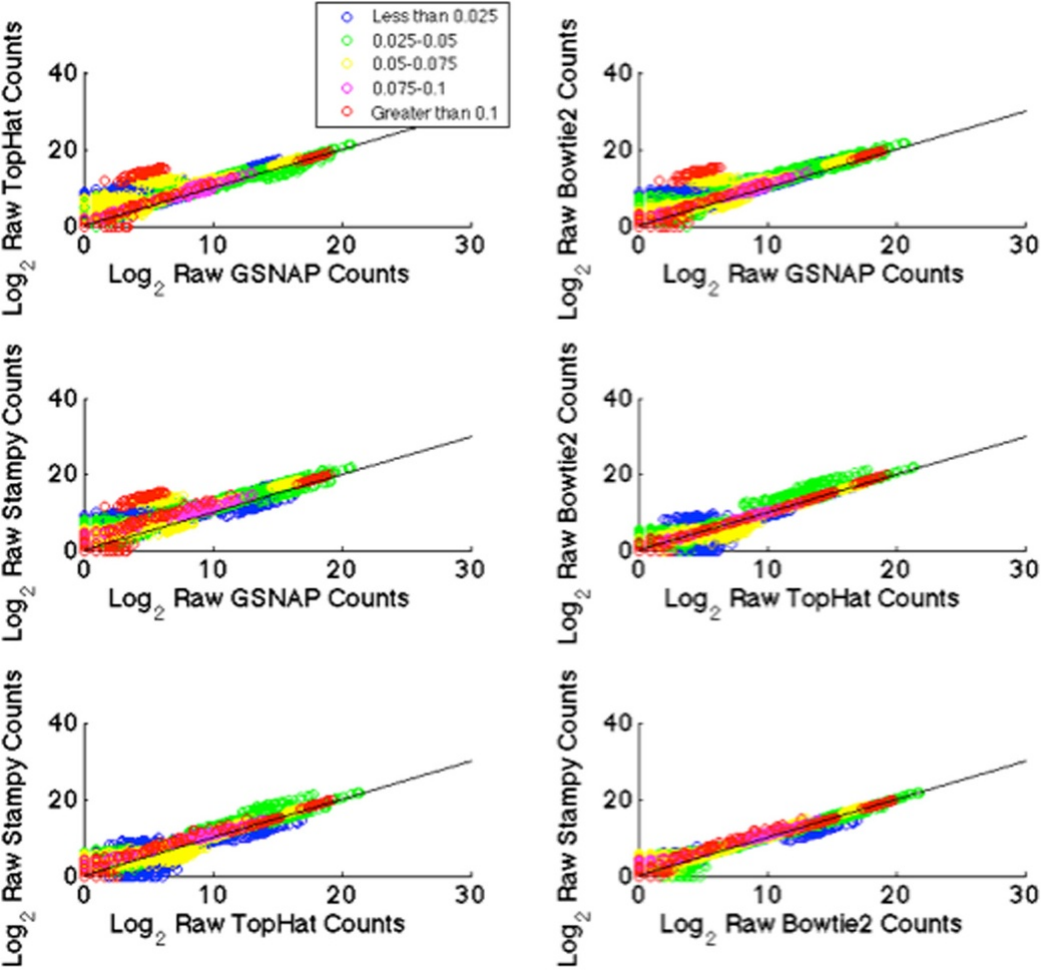
**Read Count Comparison by Evolutionary Distance.** Each scatter plot shown compares the number of reads assigned to genes by each of the mapping methods, stratified by evolutionary distance. Each panel shows a pairwise comparison of read counts between two methods. Each point indicates a particular gene in a single sample, the *l* *o* *g*_2_ raw read count in two methods. Points above the diagonal indicate higher read counts in the Y-axis method, while points below indicate higher read counts in the X-axis method.

Using the orthologous genes stratified by evolutionary distance described above, we compared the number of reads mapping to homologous human genes of varying evolutionary distance by the four mapping methods Bowtie2, Stampy, TopHat2, and GSNAP. Figure 5 shows read count comparisons between methods for 453 orthologous genes, colored by Jukes-Cantor evolutionary distance. Points above the diagonal indicate a higher read count from the mapping method indicated on the Y-axis, while points below the diagonal indicate a higher read count from the mapping method indicated on the X-axis. Similar results comparing mapping methods Bowtie2-G, Stampy-G, TopHat2, and GSNAP are shown in Additional file 1: Figure S3. Most notable is the read count differences between GSNAP with the others. All three methods obtain higher read counts for all evolutionary distances. As with the differences in mapping rates, these differences are likely due to the constraints GSNAP places on alignments - the requirement of 14 base identical stretches, and the allowance of a single gap or splice site. We see strong concordance in read counts between Bowtie2 and Stampy, with Bowtie2 obtaining slightly more read counts for more conserved genes, and Stampy obtaining slightly higher read counts for less conserved genes. TopHat2 and Bowtie2 also show strong concordance, with fairly even “spread” of read count changes. TopHat2 and Stampy are similar, with Stampy obtaining slightly higher gene counts for the most divergent genes. These results are in accordance with the mapping rate differences.

## Conclusions

We present a comparison of reference-based mapping methods for mapping Non-Human Primate RNA-Seq data to a human reference. Four different mapping approaches against the reference genome, and two mapping approaches against the reference transcriptome were assessed using mapping rates, mapping locations, detected transcripts, correlation of gene expression, and differential expression analysis. Table 4 shows a summary of our comparison of the reference-based mapping methods, when default parameter settings are used. We show that when aligning RNA-Seq reads to a surrogate reference, it is important to consider the intricate details of the methods used. We have found that the use of shorter seeds, allowance of mismatches within seeds, and the allowance for alignment gaps in addition to splice junctions are essential for sensitive read mapping. Specifically, a seed length of approximately 15 bp, allowing a single mismatch within seeds, and allowing for at least 2 gaps and/or splice junctions in spliced alignments will facilitate very sensitive read mapping. When utilizing the default behaviors of the methods compared here for mapping NHP data to a human reference, we recommend Stampy for maximum sensitivity within known genes. However, it should be noted that all methods have adjustable alignment parameters, and in most cases could be optimized for more sensitive alignment. With the proper parameter settings, each may achieve suitable sensitivity for reference-based mapping of NHP species. We also point out that a major limitation of reference-based mapping of NHP data to a human reference will not identify all genes that may be implicated in a particular phenotype. This will likely depend on the evolutionary distance between species, and on the conservation of the genes of interest. However, for exploratory purposes and hypothesis generation, reference-based mapping of NHP data may have great utility.

**Table 4:** **Summary of comparison results**

Method	Mapping	Intragenic	Detected	Detected	Gene
	Rate	mapping rate	transcripts	divergent transcripts	Expression***r*** ^2^
Transcriptome Mapping with Bowtie2	◐		◐	◐	
Transcriptome Mapping with Stampy	◐				
Genome Mapping with Bowtie2			◐	◐	◐
Genome Mapping with Stampy			◐	◐	
Genome Mapping with TopHat2	◐	◐	◐	◐	◐
Genome Mapping with GSNAP			◐	◐	◐

- Good, ◐ - Better,  - Best.

## Methods

### Study samples

We obtained data from 12 adult male colony-bred yellow baboons (*Papio cynocephalus*), inoculated with five different levels of *Streptococcus pneumoniae*, a bacterial pathogen causing pneumonia [25]. Animals were housed in the Duke University Vivarium (Durham, NC) and handled in accordance with American Association for Accreditation of Laboratory Animal Care guidelines. The experimental protocol was approved by the Duke University Institutional Animal Care and Use Committee.

The bacterial doses administered to the participants included 10 ^9^ colony forming units (CFU) (n = 4), 10 ^8^ CFU (n = 3), 10 ^7^ CFU (n = 1), 10 ^6^ CFU (n = 1), and 0 CFU (n = 3). Peripheral blood samples for gene expression analysis were taken at five different time points - immediately before inoculation, and 6, 24, 48, and 168 hours following inoculation. Antibiotics were administered immediately following the 48 hour time point. Each participant was evaluated to determine clinical pneumonia status using pre-determined criteria. A participant was classified as developing clinical pneumonia if each of the three following conditions were met:

-  A white blood cell count of greater than 15,400, less than 3,400, a 2–fold change from baseline measurement, or greater than or equal to 90% neutrophilia at 24 or 48 hours.
-  A positive culture of *Streptococcus pneumoniae* from Bronchoalveolar Lavage (BAL) or blood samples at 48 hours.
-  Any one or more of the following at 24 or 48 hours:
  - Heart rate of greater than 100 bpm
  - A 25% or greater increase in respiratory rate from baseline
  - Positive indication of infiltrate on a chest X-ray
  - Decreased Activity
  - Decreased Food Intake
  - A fever of 38.2 degrees Celsius or greater
  - Cough
  - Nasal Discharge

Three of the 60 samples did not meet RNA quality standards. Table 2 shows the participants meeting clinical criteria at 48 hours. Sequence data at baseline for all participants has been deposited in NCBI’s Gene Expression Omnibus [26] and are accessible through GEO Series accession number GSE57485 (http://www.ncbi.nlm.nih.gov/geo/query/acc.cgi?acc=GSE57485).

### RNA isolation and library preparation

Samples were collected in PreAnalytiX PAXgene Blood RNA collection tubes, and total RNA was isolated using the PreAnalytiX PAXgene Blood RNA miRNA isolation kit. RNA quality was assessed using Agilent Bioanalyzer and Nanodrop spectrophotometry, and samples with RNA integrity number greater than or equal to 7, and greater than or equal to 1 microgram of RNA were deemed sufficient for RNA-Seq. Abundant globin transcripts were depleted with the GlobinClear Globin RNA Reduction for RNA-Seq protocol. The fragment library was prepared with the Illumina TruSeq RNA Seq protocol, and Illumina HiSeq RNA Sequencing was performed, run in 6-plex per flow cell lane, obtaining 50 bp paired-end reads.

### Read quality control and trimming

Read quality analysis was performed on the raw data using FastQC version 0.10.1 [27]. Quality trimming and adapter clipping were performed using Trimmomatic version 0.25 [28], trimming trailing bases below quality 20, clipping Illumina adapters, and discarding clipped reads shorter than 25 bp. FastQC was used to re-assess the integrity of the clipped reads prior to subsequent mapping and analysis. Reads whose mates were discarded due to quality trimming and length constraints were removed from the fastq files used for mapping. Table 2 shows the number of read pairs in millions for each sample, before and after quality trimming.

### Read mapping

The Illumina iGenomes UCSC hg19 human reference genome and annotation was used as a reference, downloaded March 2013. To generate a fasta file of transcripts for a reference transcriptome, the BEDTools “getfasta” utility version 2.17.0 [29] was used to extract the transcript sequences specified by coordinates in the Ensembl GTF file, from the reference genome sequence. Both the Ensemble reference annotation and genomic sequence files were those included in the iGenomes download. Clipped reads were mapped to the hg19 genome and transcriptome using Bowtie2 version 2.0.6. [12], and Stampy version 1.0.17. [11]. Clipped reads were mapped to the hg19 human genome using Tophat version 2.0.7 [19], and GMAP (GSNAP) version 2013-03-12 [18]. Default parameter settings were used for all methods. SAM/BAM conversions, sorting, indexing, and marking of PCR duplicates were performed with SAMtools version 0.1.18 [30] and Picard version 1.83 [31].

### Quantification and normalization

Read counts for each transcript were obtained with HTSeq [32], specifically the intersection-nonempty mode of htseq-count. When quantifying expression for differential expression analysis, only the primary alignments for each read were counted by HTSeq. For the purpose of this manuscript, we investigated the default sensitivity of each mapping method when mapping NHP RNA-seq data to a human reference, and so the reporting of suboptimal alignments was not critical. Conditional Quantile Normalization (CQN) was used to obtain normalized gene expression estimates [33].

### Mapping comparisons

To compare the reference-based mapping methods, we examined several mapping metrics including mapping rates, mapping locations, transcripts detected, mate pair concordance, and coverage over transcripts. All mapping metrics were computed with RNA-SeQC version 1.1.7 [22], for the entire gene set, as well as subsets of genes in Gene Ontology [24] functional groupings, and evolutionary distance groupings. See the corresponding methods sections for more details on the generation of these gene lists.

#### Functional groups

To assess the number of detected genes within different functional groupings, we took the sets of genes present within each of the 23 top-level biological process ontology terms to generate 23 functional group gene lists. BEDtools version 2.17.0 [29] was used to select reads mapping to each gene list from each of the BAM files, which were then analyzed with RNA-SeQC to determine the number of detected genes.

#### Evolutionary distance groups

To assess the number of detected genes at various evolutionary distances, we obtained the mRNA sequence of all olive baboon (*Papio anubis*) RefSeq genes from the UCSC genome browser, and determined the human ortholog and evolutionary distance of each reference baboon gene. Orthologs were found by performing a BLASTN [34] search against the human transcriptome, and taking the top hit by percent identity. Jukes-Cantor distances [35] were then computed between the orthologous sequences. Five subset gene lists were created using evolutionary distance: greater than or equal to 0.1 (n = 19), less than 0.1 and greater than or equal to 0.075 (n = 20), less than 0.075 and greater than or equal to 0.05 (n = 62), less than 0.05 and greater than or equal to 0.025 (n = 202), and less than 0.025 (n = 155). As with the functional gene lists, BEDtools version 2.17.0 [29] was used to select reads mapping to each gene list from each of the BAM files.

#### Differential expression analysis

For each of the four mapping methods, we determined the differentially expressed transcripts using edgeR [36]. The CQN package was used to compute gene-specific correction factors, or offsets, and these offsets were provided to the glm functions of edgeR to correct for GC content and transcript length biases. This procedure is detailed in the cqn manual [37]. Across methods, we report the number of shared genes exhibiting significant differential expression (p <0.05 after Bonferonni correction) between healthy participants, and participants with clinical pneumonia at maximal symptoms (48 hours). A 4-way venn diagram web tool was used to generate figures of overlapping DE genes [38].

### Computational resources

All read mapping and quality control analyses were performed on the Duke Shared Computing Resource, a cluster of Intel x86 compute notes running Linux. Each sample using each mapping method was run on a single node with 16 GB of memory.

## Electronic supplementary material

Additional file 1: **Supplementary Figures illustrating all pairwise Spearman correlations between samples (Figure S1), boxplots of Spearman correlations between baseline samples (Figure S2), and Read count comparisons stratified by evolutionary distance for genome mappings with Bowtie2 and Stampy (Figure S3).** (DOCX 86 KB)

## Abbreviations

BAL: Bronchoalveolar lavage
BWT: Burrows-wheeler transform
CFU: Colony forming unit
DE: Differentially expressed
NGS: Next generation sequencing
NHP: Non-human primate.
