# Supplementary material for: Comparing reference-based RNA-Seq mapping methods for non-human primate data
Source: BMC Genomics. 2014 Jul 7;15(1):570. doi: 10.1186/1471-2164-15-570 (PMC4112205; doi:10.1186/1471-2164-15-570)
Supplement: Supplementary file 1 — Additional file 1: Supplementary Figures illustrating all pairwise Spearman correlations between samples (Figure S1), boxplots of Spearman correlations between baseline samples (Figure S2), and Read count comparisons stratified by evolutionary distance for genome mappings with Bowtie2 and Stampy (Figure S3). (DOCX 86 KB) [file 12864_2013_6284_MOESM1_ESM.docx]

Additional file 1: Figure S1 – Overall Rank-based Correlation of Gene Expression

Additional file 1: Figure S1 shows a heat map of all pairwise Spearman correlations between gene expression of each sample computed with each of the four mapping methods.

Additional file 1: Figure S2 - Correlation of Gene Expression

Additional file 1: Figure S2 shows boxplots of the Spearman correlations between gene expression of baseline samples (0 hours), within each method.

Additional file 1: Figure S3 – Read Count Comparison by Evolutionary Distance

Additional file 1: Figure S3 shows scatter plots comparing the number of reads assigned to genes by each of the mapping methods, stratified by evolutionary distance. The Bowtie2 and Stampy read counts shown are from mapping to the reference genome. Each panel shows a pairwise comparison of read counts between two methods. Each point indicates a particular gene in a single sample, the log_2_ raw read count in two methods. Points above the diagonal indicate higher read counts in the Y-axis method, while points below indicate higher read counts in the X-axis method.
